# Listeriolysin O Pore-Forming Activity Is Required for ERK1/2 Phosphorylation During *Listeria monocytogenes* Infection

**DOI:** 10.3389/fimmu.2020.01146

**Published:** 2020-06-03

**Authors:** Changyong Cheng, Jing Sun, Huifei Yu, Tiantian Ma, Chiyu Guan, Huan Zeng, Xian Zhang, Zhongwei Chen, Houhui Song

**Affiliations:** Key Laboratory of Applied Technology on Green-Eco-Healthy Animal Husbandry of Zhejiang Province, China-Australia Joint Laboratory for Animal Health Big Data Analytics, Zhejiang Provincial Engineering Laboratory for Animal Health Inspection & Internet Technology, College of Animal Science and Technology & College of Veterinary Medicine of Zhejiang A&F University, Hangzhou, China

**Keywords:** *Listeria monocytogenes*, listeriolysin O (LLO), pore-forming activity, ERK1/2 phosphorylation, cholesterol-binding motif

## Abstract

Listeriolysin O (LLO) is a cholesterol-dependent cytolysin that mediates escape of *L. monocytogenes* from phagosomes and enables the bacteria to grow within the host. LLO is a versatile tool allowing *Listeria* to trigger several cellular responses. In this study, rapid phosphorylation of ERK1/2 on Caco-2 cells caused by *Listeria* infection was demonstrated to be highly dependent on LLO activity. The effect could be strongly induced by adding purified recombinant LLO alone and could be inhibited by exogenous cholesterol. Lack of the PEST sequence, known to tightly control cytotoxicity of LLO, did not affect ERK1/2 activation. However, the recombinant non-cytolytic LLO_T515AL516A_, with mutations in the cholesterol-binding motif, was unable to trigger this response. Recombinant LLO_N478AV479A_, which lacks most of the cytolytic activity, also failed to activate ERK1/2 phosphorylation, and this effect could be rescued when the protein concentration reached a cytolytic level. Infection with an LLO-deficient mutant (Δ*hly*) or the mutant complementing LLO_T515AL516A_ abrogated the capacity of the bacteria to activate ERK1/2. However, infection with the Δ*hly* mutant complementing LLO_N478AV479A_, which retained partial pore-forming ability and could grow intracellularly, was capable of triggering ERK1/2 phosphorylation. Collectively, these data suggest that ERK1/2 activation by *L. monocytogenes* depends on the permeabilization activity of LLO and more importantly correlates with the cholesterol-binding motif of LLO.

## Introduction

*Listeria monocytogenes*, a facultative intracellular pathogen that has the capacity to actively invade and multiply within mammalian cells, is the causative agent of listeriosis, which affects immunocompromised individuals, as well as pregnant women and elderly people (1, 2). *L. monocytogenes* can thrive in a variety of environments and has the remarkable ability to cross various host barriers. Owing to its environmental adaptability and unique intracellular lifestyle, this bacterium has come to the forefront as a model system to study bacterial infection biology and host-pathogen interactions (3). Listeriolysin O (LLO) is a key determinant of *L. monocytogenes* pathogenesis, mediating vacuole degradation and escape. LLO is a member of the cholesterol-dependent cytolysins (CDCs), which is the largest family of bacterial pore-forming toxins (PFTs) produced by many pathogenic Gram-positive bacteria (4–6). LLO is a phagosome-specific cytolysin that forms pores in host membranes and is continuously expressed throughout the intracellular lifecycle of *L. monocytogenes*. Uncontrolled expression of LLO could lead to perforation of organelles and the host plasma membrane from the inside of the cell, causing cell death and destruction of the intracellular niche of *L. monocytogenes*, and thereby exposing the bacteria to the host immune system (5, 7, 8). Therefore, *L. monocytogenes* tightly controls synthesis and activity of LLO to disrupt vacuolar membranes without killing host cells. It is well-established that *L. monocytogenes* mutants with increased LLO expression or activity efficiently escape from vacuoles but are less virulent because they over-toxic to host cells, thereby destroying their intracellular niche (9). LLO is the only cytolysin of the PFTs that is made by an intracellular pathogen. As a result, LLO has an incontrovertible acidic pH optimum and can be denatured at neutral pH to restrict its cytolytic activity (10). Moreover, the PEST-like sequence of LLO near its N-terminus that reduces the intracellular toxicity of this cytolysin is necessary for *L. monocytogenes* to better survive intracellularly following escape from phagocytic vacuoles (6, 11).

Exposure to PFTs leads to plasma membrane damage and cell death, and the LLO-induced pore-forming effect that results in rapid Ca^2+^ influx and K^+^ efflux can also trigger different categories of cellular responses during bacterial infection (12). These responses include modulation of mitogen-activated protein kinase (MAPK) (13–15), SUMOylation dysregulation (16), endoplasmic reticulum stress (17), mitochondrial fragmentation (18), inflammasome activation (19) and histone modification (20). The MAPK signaling transduction pathway, one of the most important regulatory mechanisms in eukaryotic cells and essential for the host immune response, can be manipulated by bacterial pathogens to their advantage (21, 22). Activated MAPK translocates to the nucleus to activate, by phosphorylation, proteins required for transcription of genes, including proinflammatory signaling molecules. Three different mammalian MAPK cascades have been described to date, and are named according to their MAPK components: extracellular signal-regulated kinase 1 and 2 (ERK1/2), as well as c-Jun N-terminal kinase (JNK) and p38, with all three activated by hierarchical phosphorylation (23).

Given the importance of MAPK signaling pathways in regulating immune responses, it is not surprising that many bacterial pathogens have developed mechanisms to directly or indirectly modulate MAPK activation or inhibition (24). These pathogens often use effector proteins to manipulate MAPK pathways and allow the bacteria to establish infection within the host (21). Employing a model of the blood-cerebrospinal fluid barrier based on human choroid plexus epithelial papilloma (HIBCPP) cells, a previous study showed that infection with *L. monocytogenes* triggers activation of ERK1/2 and p38 signaling, and such cellular response is required for *L. monocytogenes* infection (22, 25). Modulation of MAPK pathway signaling by LLO during *L. monocytogenes* infection has been described in various host cell lines. Infection of epithelial cells by *L. monocytogenes* could activate phosphorylation of MAPK kinases through the action of LLO, and this was essential for invasion of *Listeria* into host cells (13–15). On the contrary, LLO also contributes to inhibition of MAPK signaling pathway activation and infection-associated abortion by dephosphorylation of MAPK family proteins during *L. monocytogenes* infection in trophoblast giant cells (26). Unlike other CDCs, LLO displays unique characteristics that limit its cytotoxicity. However, correlation between the pore-forming activity of LLO and the ability to manipulate MAPK pathways is unclear, and the detailed mechanistic contribution of LLO in MAPK pathway signaling needs further study.

In the present study, the roles of the critical domains of LLO that determine pore-forming activity involved in the phosphorylation of ERK1/2 MAP kinases during *L. monocytogenes* infection of human intestinal epithelial cells were investigated. Our data suggest that permeabilization activity on host cell membranes by the amino acid pair Thr515-Leu516 of LLO is critical to activate *L. monocytogenes* infection-induced ERK1/2 phosphorylation. Furthermore, adding the purified LLO alone at a very low cytotoxic concentration is sufficient to induce this cellular response, while mutations in the cholesterol-binding motif render LLO incapable of activating ERK1/2 signaling.

## Materials and Methods

### Bacterial Strains and Culture Conditions

The strains *L. monocytogenes* EGD-e, Δ*hly*, CΔ*hly*, CΔ*hly*_N478AV479A_, and CΔ*hly*_T515AL479A_, and *E. coli* DH5α and Rosetta were used in this study. The gene deletion mutant Δ*hly* was generated from parental strain EGD-e using the homologous recombination method (27). All complemented strains were derived from Δ*hly*. Mutants of the *hly* gene were cloned into the integration plasmid pIMK2 driven by the *hly* promoter and then introduced into Δ*hly* background. All resulting mutations were confirmed by sequencing. All strains of *Listeria* were cultured in brain heart infusion (BHI) broth (Thermo Fisher Scientific, Waltham, USA) at 37°C with shaking or on BHI broth containing 1.5% agar (Sangon, Shanghai, China). *E. coli* strains were grown at 37°C in Luria-Bertani broth (LB) (Thermo Fisher Scientific) or on LB broth containing 1.5% agar.

### Overexpression and Purification of His-Tagged LLO Proteins From *E. coli*

Recombinant LLO proteins used in this study were expressed as fusion proteins to the N-terminal His-tag using pET30a(+) as the expression vector (4). Briefly, the *hly* gene from EGD-e genome was amplified and inserted into the pET30a(+) vector, and finally transformed into Rosetta competent cells. *E. coli* cells harboring the recombinant plasmids were grown in 500 mL LB medium supplemented with 50 μg/mL kanamycin at 37°C until the cultures reached 0.8–1.0 at OD_600nm_. Isopropyl β-D-1-thiogalactopyranoside (IPTG) was then added to a final concentration of 0.4 mM to induce expression of the recombinant proteins for additional 4 h. The His-tagged fusion proteins were purified using the nickel-chelated affinity column chromatography. For generation of the mutants (LLO_N478AV479A_ and LLO_T515AL516A_), the QuikChange Site-Directed Mutagenesis kit (Agilent Technologies, Palo Alto, USA) was used according to the instructions. All mutant constructs were sequenced to ensure that only the desired single mutations had been incorporated correctly. These LLO mutant proteins were expressed and purified as described above.

### LLO-Mediated Hemolytic Assay

Measurement of LLO-associated hemolytic activity was performed as previously described (4, 28). Briefly, strains of *L. monocytogenes* were grown for 12 h with shaking in BHI broth at 37°C. All cultures were adjusted to an OD_600_ of 1.0 before supernatant protein samples were collected. Hemolytic activity was measured based on lysis of sheep red blood cells (SRBCs) by secreted LLO from culture supernatants. Specifically, culture supernatant (50 μL) was diluted in hemolysis buffer (10 mM PBS, pH 5.5 or 7.4, 150 mM NaCl, 1 mM DTT) in a final volume of 50 μL, and equilibrated to 37°C for 10 min. Next, 100 μL PBS-washed intact SRBCs (5%) were added to each sample and incubated at 37°C for 30 min. Samples were centrifuged and supernatants analyzed for hemoglobin absorption at 550 nm. For hemolysis determination of recombinant proteins, purified LLO or mutant LLO protein (LLO_N478AV479A_ or LLO_T515AL516A_) expressed in *E. coli* was serially diluted in hemolysis buffer, then mixed with an equal volume of 5% SRBC and the hemolytic activity was determined as described above. The values corresponding to the reciprocal of the dilution of culture supernatant required to lyse 5% SRBCs were used to compare the hemolytic activities in the different supernatants. Erythrocytes incubated with 1% Triton X-100 or PBS served to determine the maximum (100%) and minimum (0%) hemolytic activity, respectively.

### Immunoblotting

Cytoplasmic cell extracts were separated by SDS-PAGE and transferred to polyvinylidene difluoride (PVDF) membranes for western blotting. Membranes were incubated with p44/42 MAPK (ERK1/2) rabbit monoclonal antibody (Cell Signaling Technology, Danvers, USA), Phospho-p44/42 MAPK (ERK1/2) rabbit monoclonal antibody (Cell Signaling Technology), or anti β-actin antibody (Sigma-Aldrich, St. Louis, USA) at 4°C overnight, as appropriate. After washing with Tris-buffered saline (TBS) containing 0.02% (v/v) Tween 20, membranes were incubated with horseradish peroxidase-conjugated secondary antibody (Sigma-Aldrich) at 37°C for 1 h. Immunoreactions were then visualized using the enhanced chemiluminescence detection system (UVP Inc., Upland, USA).

### Cell Fractionation and Protein Localization of LLO

Western blotting was employed to analyze the changes in expression of LLO as previously described (29). Overnight cultures of *L. monocytogenes* were diluted into 200 mL fresh BHI broth, and bacteria were grown to stationary phase. For isolation of secreted proteins, bacterial cells were pelleted by centrifugation at 13,000 ***g*** for 20 min at 4°C, and the resulting supernatant was collected and passed through a 0.22 μm polyethersulfone membrane filter (Thermo Fisher Scientific). Trichloroacetic acid (TCA) was added to the supernatant to a final concentration of 10%. Proteins were TCA-precipitated on ice overnight and washed with ice-cold acetone. The washed precipitates were resuspended in SDS-PAGE sample buffer (5% SDS, 10% glycerol, and 50 mM Tris–HCl, pH 6.8), boiled for 6 min and stored at −20°C before electrophoresis. For isolation of total cell proteins, bacterial pellets were resuspended in 1 mL extraction solution (2% Triton X-100, 1% SDS, 100 mM NaCl, 10 mM Tris–HCl, 1 mM EDTA, pH 8.0). One gram of glass beads (G8772, Sigma-Aldrich) was added and samples were lysed using a Precelly 24 homogenizer (Bertin, Provence, France) at 6,000 rpm for 30 s with intermittent cooling for 30 s (three cycles in total). Samples were then centrifuged at 12,000 rpm for 15 min, and the supernatant was retained as the whole cell extract. Protein samples were separated through a 12% SDS-PAGE gel and were immunoblotted with α-LLO, or α-GAPDH antibodies. GAPDH was used as an internal control.

### Propidium Iodide Assay of Membrane Integrity

Caco-2 cells were plated at 1.0 × 10^6^ cells per well in 12-well tissue culture plates with glass coverslips and were cultured for 12 h. Cells were infected with bacteria [multiplicity of infection (MOI) 10:1] or treated with various concentrations of LLO (or its variants) diluted in serum-free DMEM for 3 h or 30 min at 37°C, respectively. Cells were then washed with PBS and incubated with 0.4 mg/ml propidium iodide (Sigma-Aldrich) at 37°C for 10 min before fixing with 4% paraformaldehyde and counterstaining with DAPI (4′,6-diamidino-2-phenylindole) (Thermo Fisher Scientific). Cells were imaged on a ZEISS LSM510 confocal microscope equipped with a ×10 objective.

### Proliferation in RAW264.7 Macrophages

Stationary *L. monocytogenes* were washed and re-suspended in 10 mM PBS (pH 7.4). Monolayers of RAW264.7 cells cultured in DMEM (Thermo Fisher Scientific) containing 10% FBS (Hyclone, Chicago, USA) were infected with bacteria at an MOI of 0.05. After incubation for 30 min, infected cells were washed twice with PBS and incubated in DMEM containing 50 μg/mL gentamicin for additional 30 min to kill extracellular bacteria. At 2, 6, 12, or 18 h post infection, cells were lysed by adding 1 mL ice-cold sterile distilled water and lysates were diluted 10-fold for enumeration of viable bacteria on BHI agar plates.

### Plaque Assay in L929 Fibroblast Cells

The plaque assay was carried out by conventional methods (4). Briefly, murine L929 fibroblast cell monolayers were maintained in high-glucose DMEM containing FBS (Hyclone) and 2 mM L-glutamine. Cells were seeded at 1 × 10^6^ cells per well in 6-well plates and infected with *L. monocytogenes* at an MOI of 1:50 for 1 h at 37°C and 5% CO_2_. Extracellular bacteria were killed with 50 μg/mL gentamicin for 60 min, and the cells washed three times with 10 mM PBS (pH 7.4) and then overlaid with 3 mL medium plus 0.7% agarose and 10 μg/mL gentamicin. Following a 72-h incubation at 37°C, cells were fixed with paraformaldehyde (4% in PBS for 20 min) and stained with crystal violet. Plaque diameter for each bacterial strain was measured using Adobe Photoshop software. The plaque size of wild-type strain EGD-e was set as 100% and data are shown as mean ± SD.

### Cytotoxicity Detection

Cytotoxicity was detected based on lactate dehydrogenase (LDH) release from J774 macrophages following bacterial infection and using the CytoTox 96 non-radioactive cytotoxicity assay kit according to the manufacturer's instructions (Promega, Wisconsin, USA), as previously described (4, 30). Overnight cultures of *L. monocytogenes* were deposited onto J774 cells at a MOI of 10 and incubated for 30 min at 37°C and 5% CO_2_. Culture medium with or without 50 μg/mL gentamicin was then added. To determine maximum LDH release, 100 μL lysis buffer was added to triplicate-infected wells 45 min prior to LDH measurement. At the indicated infection times (2, 4, and 6 h), cells were centrifuged at 250 ***g*** for 5 min, and the supernatant was removed and used for the LDH assay. The supernatant was incubated for 30 min with 50 μL substrate mix prior to the addition of 50 μL stop solution. Absorption of the samples at 490 nm was then measured using a Synergy H1 micro-plate reader (BioTek, Winooski, USA). The experimental design included 3-wells containing only DMEM to account for background absorption, as well as 3-wells containing uninfected J774 cells to measure spontaneous LDH release. After background correction, the percent cytotoxicity was calculated as follows: cytotoxicity % = [(experimental LDH release–spontaneous LDH release)/(maximum LDH release–spontaneous LDH release)] × 100.

### Virulence in the Mouse Model

Wild-type and mutant strains of *L. monocytogenes* were tested for their ability to be recovered from mouse organs (livers and spleens). Briefly, ICR mice (female, 18–22 g) (eight mice per group) were inoculated intraperitoneally with 10^6^ CFU of each strain. At 24 and 48 h post-infection, mice were sacrificed, and livers and spleens were removed and individually homogenized in 10 mM PBS (pH 7.4). Surviving bacteria were enumerated by plating serial dilutions of homogenates on BHI agar plates.

### Statistical Analysis

All experiments were repeated at least three times. Data were analyzed using the two-tailed homoscedastic Student's *t*-test. *P*-values < 0.05 were considered statistically significant.

### Ethics Statement

All animal care and use protocols were performed in accordance with the Regulations for the Administration of Affairs Concerning Experimental Animals approved by the State Council of People's Republic of China. The protocol was approved by the Institutional Animal Care and Use Committee of Science Technology Department of Zhejiang Province (Permit Number: SYXK-2018-0010). All the *L. monocytogenes*-involved experiments in our study were conducted at Biosafety Level 2 (BSL-2) laboratory.

## Results

### LLO Is Critical for Induction of ERK1/2 Phosphorylation in Response to *L. monocytogenes* Infection in Caco-2 Cells

**Figure 1:**
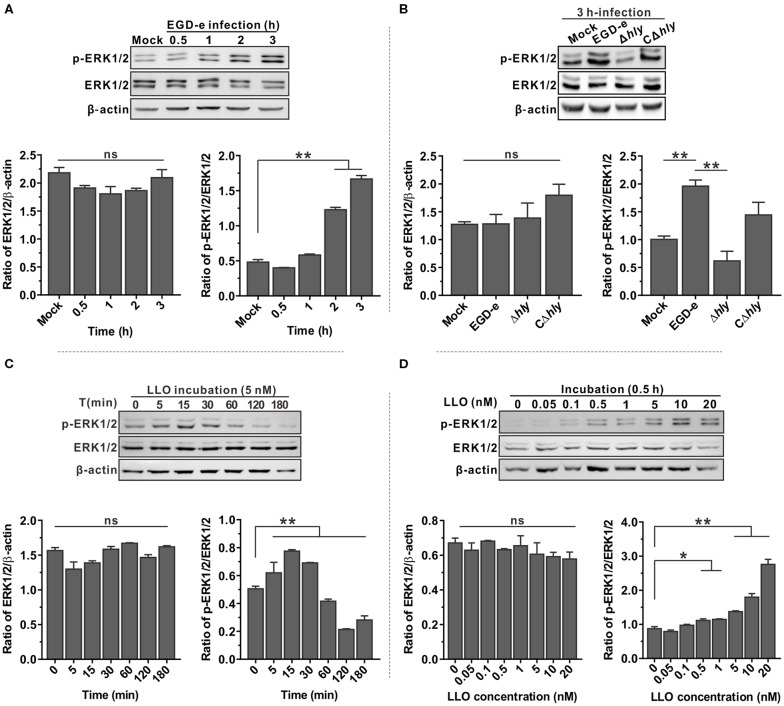
ERK1/2 phosphorylation triggered by *L. monocytogenes* infection in Caco-2 cells is LLO-dependent. **(A,B)** Expression of MAP kinases ERK1 and ERK2 in Caco-2 human epithelial cells infected with *L. monocytogenes* wild-type EGD-e **(A)** or the *hly* deletion and complemented mutants **(B)** for the indicated time points. Expression of the indicated proteins was detected by immunoblotting, with β-actin used as an internal control. Phosphorylated ERK1/2 is designated p-ERK1/2. **(C,D)** Expression of ERK1/2 in Caco-2 cells treated with purified recombinant LLO at different time points **(C)** and with various concentrations of protein **(D)**. Intensity of ERK1/2 protein levels was measured using Quantity One software (Bio-Rad), and total ERK1/2 and phosphorylated ERK1/2 were normalized by β-actin and total ERK1/2 values, respectively. All values represent the means ± SD of three independent experiments. *P < 0.05; **P < 0.01; ns, not significant.

Since the MAPK ERK1/2 pathway is involved in infection of host cells by bacterial pathogens, phosphorylation of ERK1/2 in Caco-2 cells after infection with *L. monocytogenes* was analyzed by immunoblotting and compared with the uninfected control. Phosphorylated ERK1/2 (p-ERK1/2) was significantly activated by bacterial infection in a time-dependent manner (Figure 1A). However, the total amounts of ERK1/2 protein did not significantly change during infection with *L. monocytogenes*. The mechanism by which LLO activates ERK1/2 in host cells was then investigated. A mutant lacking the *hly* gene was unable to induce ERK1/2 phosphorylation during infection, and this compromised ability could be partially restored by complementation of a functional LLO into the *hly*-deleted mutant (Δ*hly*) (Figure 1B). Moreover, Caco-2 cells incubated with as little as ~1.5 hemolytic units (5 nM, according to the hemolytic activity assay in this study) of the purified LLO showed a more rapid phosphorylated ERK1/2 activation starting at 5 min incubation with a maximum at 15 and 30 min, and decreasing as the incubation time continued (Figure 1C). The activation effect of LLO on ERK1/2 phosphorylation was concentration-dependent, and a very low concentration of 0.5 nM was sufficient to activate ERK1/2 phosphorylation (Figure 1D). Taken together, these results established a critical role for LLO in the activation of p-ERK1/2 during *L. monocytogenes* infection of epithelial cells.

### Pore-Forming Activity of LLO Is Required for LLO-Mediated ERK1/2 Phosphorylation

**Figure 2:**
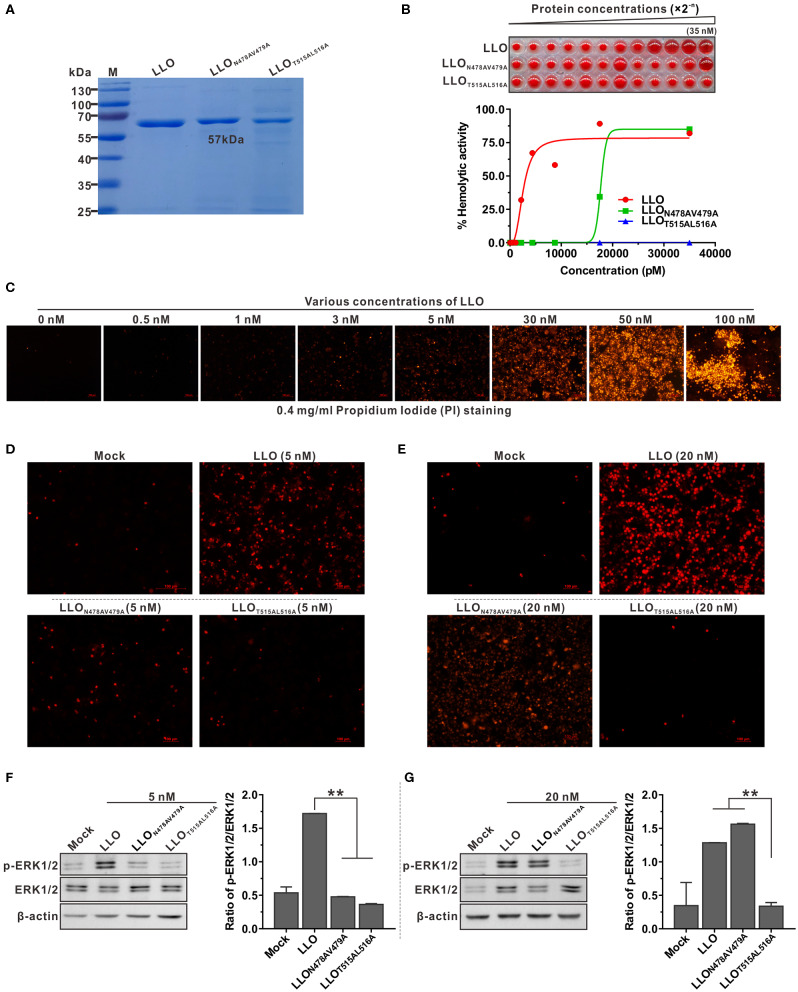
Pore-forming activity is required for LLO-triggered ERK1/2 phosphorylation in Caco-2 cells. **(A,B)** Purification of the recombinant LLO and its variants (LLO_T515AL516A_ and LLO_N478AV479A_) expressed in *E. coli* **(A)** and the hemolytic activity of these proteins at various concentrations (0–35 nM) **(B)**. Erythrocytes incubated with 1% Triton X-100 or PBS served to determine the maximum (100%) and minimum (0%) hemolytic activity, respectively. **(C–E)** Membrane integrity of Caco-2 cells treated with various concentrations of wild-type LLO **(C)** and treated with LLO variants (LLO_T515AL516A_ and LLO_N478AV479A_) at 5 nM **(D)** and 20 nM. **(E)** Cells were treated with sub-lytic and lytic concentrations of recombinant LLO and membrane integrity was assessed by labeling with 0.4 mg/ml propidium iodide. **(F,G)** ERK1/2 phosphorylation in Caco-2 cells treated with LLO or its variants (LLO_T515AL516A_ and LLO_N478AV479A_) at the sub-lytic concentration (5 nM) **(F)** or at the lytic concentration (20 nM) **(G)**. Values in **(B,F,G)** represent the mean ± SD of three independent experiments. **P < 0.01; ns, not significant.

To explore the correlation between pore-forming activity of LLO and its ability to activate ERK1/2 phosphorylation, two double-amino-acid mutations were introduced into the wild-type LLO. The resulting LLO mutant proteins (LLO_T515AL516A_ and LLO_N478AV479A_) were purified (Figure 2A) and used to investigate the effect of the mutations on ERK1/2 phosphorylation. The hemolysis assay showed that recombinant LLO_T515AL516A_ with mutations in the cholesterol-binding motif completely lost its ability to lyse erythrocytes even at high concentrations (Figure 2B). We previously showed that Asn478 and Val479 are key residues required for LLO hemolytic activity (4). In agreement with this, recombinant LLO_N478AV479A_ was completely impaired in cytolytic ability within a certain concentration range; however this could be fully restored by increasing the protein concentration to ~20 nM (Figure 2B). The ability of recombinant LLO and its variants to induce permeabilization of Caco-2 cells was further examined using propidium iodide (PI), a membrane-impermeable dye for nucleic acids. Consistently, recombinant wild-type LLO at a concentration of 5 nM was sufficient to cause obvious cell damage (Figure 2C), while at this concentration LLO_N478AV479A_ caused very little cell permeabilization (Figure 2D). When the concentration was increased to 20nM, LLO_N478AV479A_ became able to cause significant membrane damage while LLO_T515AL516A_ was still incapable of causing similar damage (Figure 2E). Next, these recombinant LLO variants at the threshold concentrations of 5 or 20 nM were used to determine their effects on ERK1/2 phosphorylation during incubation with Caco-2 cells. As expected, only wild-type LLO could significantly induce ERK1/2 phosphorylation at the low concentration (5 nM) (Figure 2F), but by increasing the concentration to 20 nM, LLO_N478AV479A_ began able to activate ERK1/2 phosphorylation as efficiently as wild-type LLO (Figure 2G). However, the LLO_T515AL516A_ remained incapable of inducing this cellular response regardless of the concentrations used (Figure 2G). A threonine-leucine amino acid pair is conserved in most CDCs and mediates direct binding of these toxins to cholesterol (31). Data obtained here confirmed that the corresponding amino acid pair (Thr515/Leu516) is required for the pore-formation activity of LLO and the consequent LLO-induced ERK1/2 activation. Collectively, these findings clearly suggested an important role of the permeable activity in LLO-mediated ERK1/2 phosphorylation.

### *L. monocytogenes* Expressing Inactive LLO Are Unable to Activate ERK1/2 Phosphorylation

**Figure 3:**
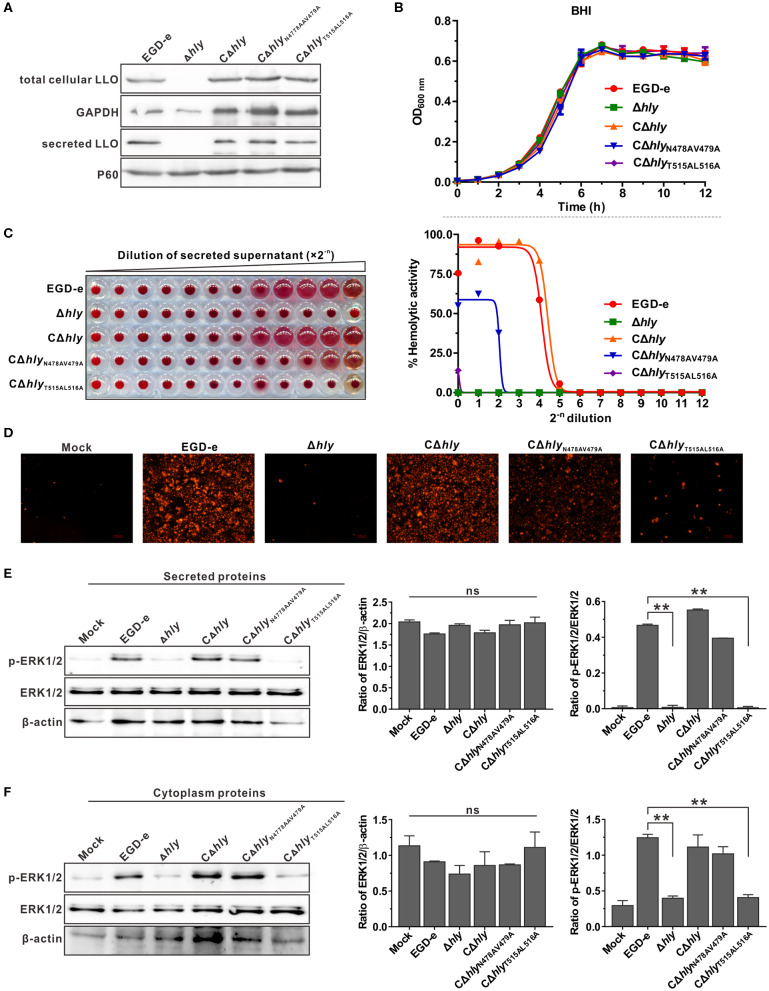
*L. monocytogenes* expressing inactive LLO were unable to activate ERK1/2 phosphorylation. **(A,B)** Secreted and cellular LLO detected by western blotting **(A)**, and *in vitro* bacterial growth **(B)** of *L. monocytogenes* wild-type EGD-e and *hly* mutant strains, Δ*hly*, CΔ*hly*, CΔ*hly*_N478AV479A_, and CΔ*hly*_T515AL516A_. **(C)** Hemolytic activity of secreted LLO from the culture supernatants of *L. monocytogenes* WT strain EGD-e and *hly* mutant strains, Δ*hly*, CΔ*hly*, CΔ*hly*_N478AV479A_, and CΔ*hly*_T515AL516A_. Erythrocytes incubated with 1% Triton X-100 or PBS served to determine the maximum (100%) and minimum (0%) hemolytic activity, respectively. **(D)** Membrane integrity of Caco-2 cells treated with bacterial secreted supernatant. Cells were treated with secreted proteins from *L. monocytogenes* WT strain EGD-e and *hly* mutant strains, Δ*hly*, CΔ*hly*, CΔ*hly*_N478AV479A_, and CΔ*hly*_T515AL516A_, and membrane integrity was assessed by labeling with 0.4 mg/ml propidium iodide. **(E,F)** Effects of secreted **(E)** and cytoplasmic **(F)** proteins from *L. monocytogenes* WT strain EGD-e and *hly* mutant strains, Δ*hly*, CΔ*hly*, CΔ*hly*_N478AV479A_, and CΔ*hly*_T515AL516A_ on ERK1/2 phosphorylation in Caco-2 cells. Values in **(B,C,E,F)** represent the mean ± SD of three independent experiments. ^**^P < 0.01; ns, not significant.

Having established that recombinant LLO_N478AV479A_ and LLO_T515AL516A_ exhibited different induction effects on ERK1/2 phosphorylation, the *L. monocytogenes* Δ*hly* mutant was complemented with wild-type LLO, LLO_N478AV479A_ or LLO_T515AL516A_ under the natural *hly* promoter using the *Listeria* integrative plasmid, pIMK2. As shown by western blotting (Figure 3A), the three complemented strains, CΔ*hly*, CΔ*hly*_N478AV479A_, and CΔ*hly*_T515AL516A_, were capable of expressing and secreting LLO or its mutant forms in comparable amounts to the wild-type in the culture supernatant, suggesting that these amino acid substitutions did not affect bacterial LLO synthesis and secretion. The mutations were also confirmed to have no effect on bacterial growth *in vitro* (Figure 3B). Importantly, hemolytic activity recorded in the secreted supernatant of the mutant CΔ*hly* with continuous dilution was comparable to that of the wild-type strain, whereas the mutant CΔ*hly*_T515AL516A_ did not exhibit any detectable hemolytic activity even in the undiluted stock supernatant, similar to the Δ*hly* mutant. The CΔ*hly*_N478AV479A_ strain retained no more than 60% wild-type hemolytic activity in the secreted supernatant at the initial dilution (Figure 3C). These results were further confirmed by membrane permeabilization assay on Caco-2 cells using PI staining (Figure 3D). Secreted and cytoplasmic proteins isolated from the above mutant strains were then used to investigate their effect on ERK1/2 phosphorylation in Caco-2 cells. The secreted and cytoplasmic LLO from wild-type and complemented strain CΔ*hly* could obviously activate phosphorylation of ERK1/2, while the corresponding protein sample from the CΔ*hly*_T515AL516A_ mutant as well as the Δ*hly* completely failed to induce such a response (Figures 3E,F). However, the secreted and cytoplasmic proteins from the mutant CΔ*hly*_N478AV479A_ were able to induce strong phosphorylation of ERK1/2 under the same conditions (Figures 3E,F), consisting with the findings obtained with the recombinant LLO variants. Taken together, although *L. monocytogenes* expressing LLO_N478AV479A_ retained incomplete hemolytic activity, it was sufficient for this bacterium to trigger ERK1/2 activation. Therefore, we can conclude that LLO-induced phosphorylation of ERK1/2 lies on the pore-forming activity of LLO that is tightly controlled during bacterial intracellular infection.

### LLO-Induced ERK1/2 Phosphorylation Is Independent of the PEST-Like Sequence but Can Be Blocked by Exogenous Cholesterol

**Figure 4:**
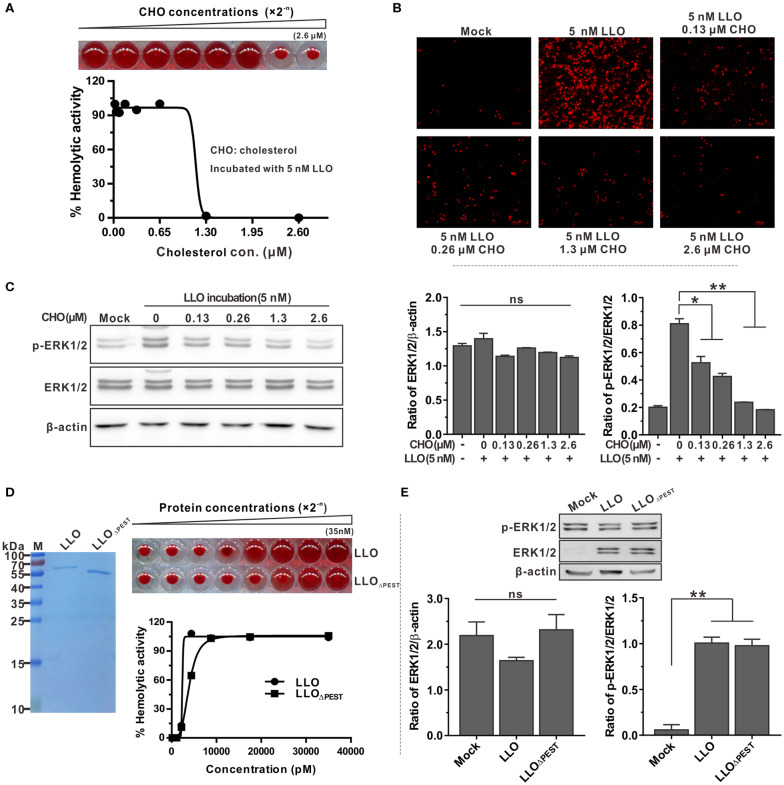
LLO-induced phosphorylation of ERK1/2 is independent of the PEST-like sequence but can be blocked by exogenous cholesterol. **(A)** Inhibitory effects of cholesterol on hemolytic activity of purified recombinant LLO. Purified LLO at a cytolytic concentration of 5 nM was pre-incubated with different concentrations of cholesterol (0–2.6 μM) and hemolytic activity was assessed by lysis of the erythrocytes. **(B)** Membrane integrity of Caco-2 cells treated with purified LLO and cholesterol. Cells were treated with 5 nM recombinant LLO that had been pre-incubated with varying amounts of cholesterol (0–2.6 μM), and the membrane integrity was assessed by labeling with 0.4 mg/ml propidium iodide. **(C)** Effects of cholesterol on LLO-induced ERK1/2 phosphorylation in Caco-2 cells. Intensity of total and phosphorylated ERK1/2 protein levels was measured using Quantity One software and normalized with the β-actin value. **(D)** Hemolytic activity of purified LLO or its variant LLO_ΔPEST_ at various concentrations (0–35 nM). Erythrocytes incubated with 1% Triton X-100 or PBS served to determine the maximum (100%) and minimum (0%) hemolytic activity, respectively. **(E)** Roles of the PEST-like sequence in LLO-induced ERK1/2 phosphorylation in Caco-2 cells. Values in **(A,C,D,E)** represent the mean ± SD of three independent experiments. *P < 0.05; **P < 0.01; ns, not significant.

The common property of all thiol-activated toxins is that pre-incubation with low amounts of exogenous cholesterol inhibits hemolytic activity as well as cytolysis of eukaryotic cells (32). This prompted us to investigate the effect of cholesterol-pretreated LLO on activation of ERK1/2 phosphorylation. As determined above, LLO at a concentration of 5 nM was sufficient to induce complete lysis of erythrocytes and ERK1/2 phosphorylation of epithelial cells. A low concentration of 1.3 μM cholesterol was sufficient to completely inhibit erythrocyte hemolysis when LLO was incubated with cholesterol before the assay (Figure 4A). This finding was further validated by the membrane permeabilization assay on Caco-2 cells using the PI staining method (Figure 4B). In addition, incubation of Caco-2 cells with LLO pretreated with varying amounts of cholesterol indicated that LLO-induced phosphorylation of ERK1/2 could be inhibited by the presence of small amounts of cholesterol, with the inhibitory effect becoming more significant as the concentration of cholesterol increased (Figure 4C). Although previous studies have documented that pretreatment of LLO with cholesterol inhibits hemolytic activity but does not interfere with the initial binding step of LLO to natural membranes (32, 33), we demonstrated that inhibition of ERK1/2 phosphorylation by pre-incubation of LLO with cholesterol was definitely due to loss of cytolytic activity but our data do not rule out an important role for cholesterol in the binding of LLO to the cell membrane. Moreover, LLO contains a unique N-terminal amino acid sequence that is absent in other CDCs, previously referred to as the PEST-like sequence (30). Removal of the PEST-like sequence had very minor effects on LLO pore-forming activity (Figure 4D) and consequently did not affect the unique properties of LLO-mediated ERK1/2 phosphorylation (Figure 4E). Collectively, these data led to the conclusion that LLO-triggered phosphorylation of ERK1/2 is independent of the PEST-like sequence of LLO but can be blocked by addition of exogenous cholesterol.

### Listeria-Induced Phosphorylation of ERK1/2 Correlates With Intracellular Infection

**Figure 5:**
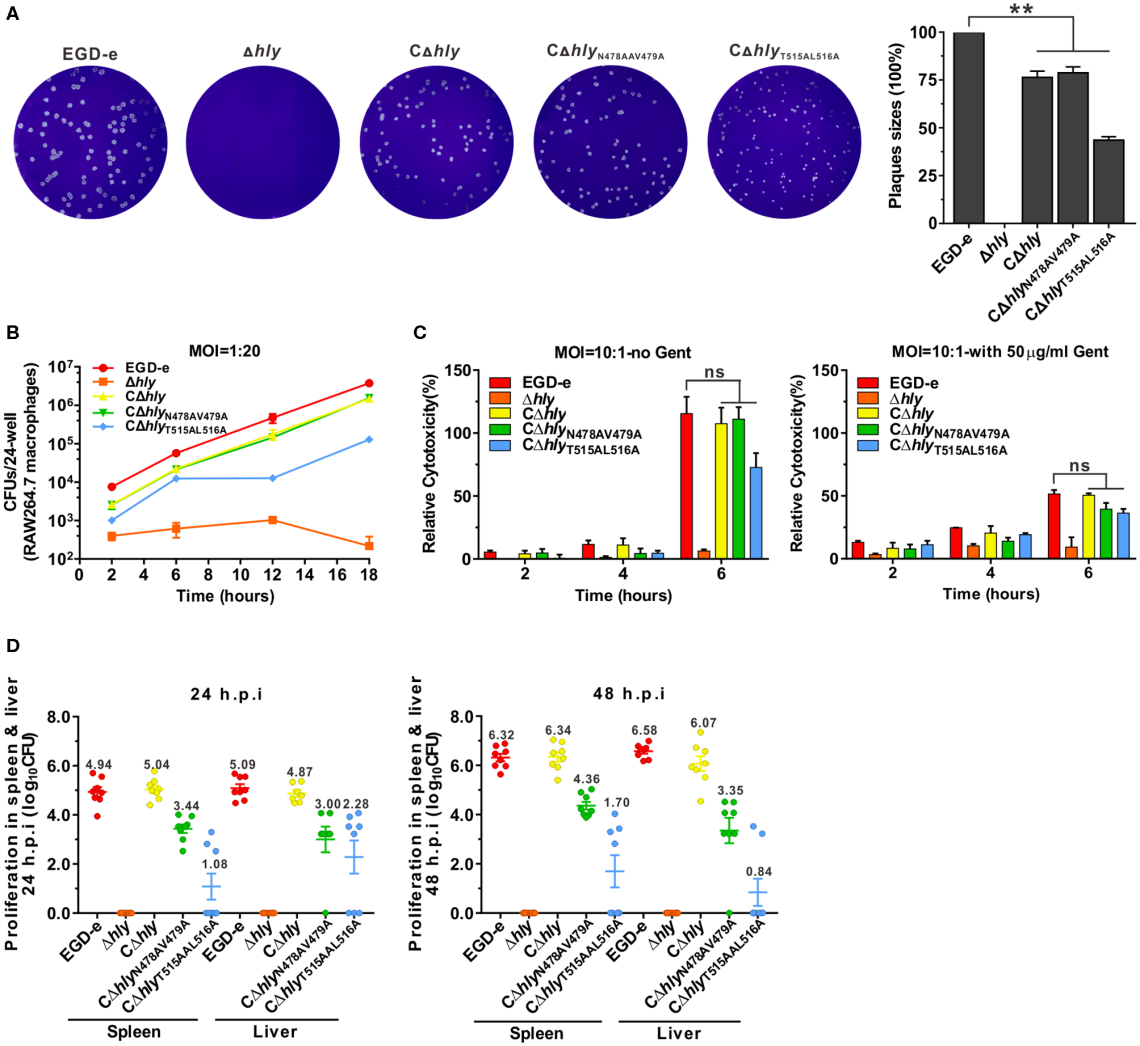
*Listeria*-induced phosphorylation of ERK1/2 correlates with intracellular infection. **(A)** Plaque sizes formed by the indicated *L. monocytogenes* mutants in L929 fibroblast cell monolayers as a percentage of the plaque size formed by wild-type bacteria. **(B)** Intracellular growth of strains of *L. monocytogenes* producing the indicated LLO proteins in murine-derived RAW264.7 macrophages. The macrophages infected with the indicated strains of *L. monocytogenes* were lysed at the indicated time points (2, 6, 12, and 18 h), and viable bacteria were serially diluted and plated on BHI agar. **(C)** Cytotoxicity of the indicated strains of *L. monocytogenes* in J774 macrophages. Lactate dehydrogenase (LDH) release into the tissue culture medium was used to monitor perforation of the host cell plasma membrane. The percentage of the maximal LDH release from monolayers of J774 macrophages infected with the indicated strains of *L. monocytogenes* at 2, 4, and 6 h post-infection in the presence or absence of gentamicin (50 μg/mL) is indicated. **(D)** Proliferation of *L. monocytogenes* in mouse organs (livers and spleens). The indicated strains were inoculated intraperitoneally into ICR mice at 5 × 10^6^ CFU. Animals were euthanized 24 and 48 h post-infection and organs (livers and spleens) were recovered and homogenized, then homogenates were serially diluted and plated on BHI agar. All values represent the mean ± SD of three independent experiments. **P < 0.01; ns, not significant.

Listeriolysin O plays an essential role in the escape of *L. monocytogenes* from the phagosome during cell-to-cell spreading. The LLO mutants with varying effects on induction of ERK1/2 activation were investigated for their ability to establish intracellular infection. Firstly, the capability of these LLO mutants to spread from cell to cell was examined by measuring the diameter of plaques formed in L929 fibroblast monolayers. Consistent with our previous findings, the partially hemolytic strain CΔ*hly*_N478AV479A_ exhibited considerable spreading efficiency compared with the CΔ*hly*, although not fully restored to the wild-type level. However, the size of plaques formed by CΔ*hly*_T515AL516A_ was significantly smaller than those resulting from infection with the other three strains, suggesting that the two cholesterol-binding sites were crucial to the spreading ability of *L. monocytogenes* (Figure 5A). In addition, CΔ*hly*_T515AL516A_ was significantly impaired in its ability to grow intracellularly in murine-derived macrophages RAW264.7, but grew far better than the LLO-deleted avirulent Δ*hly* strain that was unable to grow (Figure 5B). However, the CΔ*hly*_N478AV479A_ grew well within macrophages, with comparable efficiency to that of the CΔ*hly* or wild-type. To directly monitor the cytotoxicity of these LLO mutants, the release of a host cytosolic enzyme, lactate dehydrogenase (LDH), into the tissue culture medium from infected J774 macrophages was detected. At early time points (2 and 4 h) during infection with any of these strains, very little LDH was detected either in the presence or absence of gentamicin. At 6 h post-infection, the amount of LDH released from all the infected cells, except for the avirulent Δ*hly* strain, increased dramatically, especially in the absence of gentamicin. Surprisingly, the non-hemolytic strain CΔ*hly*_T515AL516A_ exhibited a comparable amount of LDH to the hemolytic strains (Figure 5C), demonstrating that *L. monocytogenes* expressing LLO_T515AL516A_ lacks hemolytic activity but exhibits wildtype-level cytotoxicity to host cell membranes. Moreover, the number of colony-forming units (CFU) of bacteria recovered from the spleens and livers of infected mice after 24 and 48 h of infection was markedly lower for the strains CΔ*hly*_N478AV479A_ (2–3 orders of magnitude) and CΔ*hly*_T515AL516A_ (3–5 orders of magnitude) compared with wild-type or the strain expressing wild-type LLO (Figure 5D). This indicated that the two LLO mutants were severely attenuated for virulence, and that mice infected with these mutants exhibited significantly lower bacterial burdens compared with mice infected with the wild-type strain. Combined with the different effects of these LLO mutants on ERK1/2 phosphorylation, we suggest that ERK1/2 signaling triggered by *L. monocytogenes* infection tightly correlates with the capacity of this bacteria to replicate within host cells.

## Discussion

The pore-forming toxin LLO is the only CDC produced by an intracellular pathogen and is a major virulence factor of *L. monocytogenes* involved in several stages of the intracellular lifecycle of the bacterium. Following internalization of *L. monocytogenes* into host cells, LLO disrupts the internalization vacuole, enabling the bacterium to replicate into the host cell cytosol (6). LLO is continuously produced during the intracellular infection of *L. monocytogenes*, while several processes limit its toxicity to survive better inside the host cells. Tightly controlled restriction of the activity of LLO in the internalization vacuole therefore appears to be important for *Listeria* infection. Various mechanisms responsible for restricting the activity of LLO within the host cell cytoplasm have been investigated, such as its pH sensitivity, ubiquitylation and proteasomal degradation (11). LLO is a potent signaling molecule, triggering important host cell responses via formation of a large pore complex that allows ions and small molecules to diffuse across the plasma membrane during host infection by the pathogen (9). In the present study, MAPK ERK1/2 phosphorylation in human epithelial Caco-2 cells was strongly triggered by infection with *L. monocytogenes*. This process is highly dependent on the membrane permeabilization activity of listeriolysin O. The activation effect of LLO on ERK1/2 phosphorylation was concentration-dependent, and a very low concentration of ~5 nM was sufficient to trigger this cellular response. The process could be completely inhibited by cholesterol, which blocks the membrane perforation ability of LLO. Moreover, site-directed mutagenesis of the key amino acids of LLO responsible for partial or complete abolishment of hemolytic activity resulted in different degrees of loss in the capability of LLO to induce ERK1/2 phosphorylation. Overall, these data demonstrated that *L. monocytogenes* exploits the distinctive characteristics of LLO to finely manipulate the MAPK ERK1/2 signaling pathway during intracellular infection.

Tang et al. originally identified bacterial infection as the stimulus for *L. monocytogenes*-induced tyrosine phosphorylation of the MAP kinases in HeLa cells (13). In addition, these researchers revealed LLO was the inducing agent by showing that cell-free supernatants from bacteria grown in BHI media were capable of inducing tyrosine phosphorylation (13). Subsequently, (14) confirmed that *L. monocytogenes* infection of HeLa cells enhanced activity of the Raf kinase, phosphorylation of MEK1, and phosphorylation of the two MAP kinases ERK1 and ERK2, which was most likely mediated by the action of LLO. MAPK activation by *L. monocytogenes* infection was also observed in endothelial cells. In human umbilical vein endothelial cells (HUVECs), phosphorylation of both ERK1/2 and p38, but not JNK, was detected following *Listeria* infection (22). In addition, infection with *L. monocytogenes* caused activation of the MAPKs ERK1/2 and p38 in HIBCPP cells, and such response required the *Listeria* virulence factors internalins A and B (22). However, detailed correlation between the unique properties of LLO and its effects on MAPK activation needs further clarification. In this study, activation of the JNK by *L. monocytogenes* was also not observed in either of the analyzed settings. This is consistent with the findings of Dinner and co-workers on HIBCPP cells, but is in contrast to the observations of Tang et al. in HeLa cells where JNK could also be activated by *L. monocytogenes* infection (15, 22). However, the MAPK p38 could be activated by *L. monocytogenes*, and this was also LLO-dependent (data not shown).

LLO secreted by *L. monocytogenes* perforates the host cell plasma membrane and rapidly induces a rise in intracellular Ca^2+^ and an efflux of K^+^, which have been tightly linked to activation of many signaling pathways. Calcium and calmodulin (CaM) are able to modulate activation of the MAPK pathways, including the Ras/Raf/MEK/ERK signaling pathway, and elevated concentrations of calcium can either activate or, less frequently, inhibit the ERK cascade (34–36). This study, in combination with the existing literature, illustrates the complexity of the cellular responses to LLO and how little is understood about the processes. To our knowledge, cholesterol is the only known cell membrane receptor for LLO and plays an essential role in initiating the structural transitions required for pore formation (37, 38). CDCs use cholesterol as their membrane receptor and contribute to the pathogenesis of many Gram-positive bacterial pathogens (39). Membranes that lack or are significantly depleted of cholesterol are not susceptible to the pore-forming activity of these toxins (40). A remarkably simple structure composed of a threonine-leucine pair of the CDCs functions as the cholesterol-recognition motif and is extremely important for initiating the cholesterol-dependent interaction of CDCs with membranes (31). Here, the corresponding amino acid pair (Thr515-Leu516) was confirmed to be required for LLO binding to cholesterol and subsequent pore formation, enabling LLO to trigger ERK1/2 phosphorylation. Mutants lacking LLO or expressing non-toxic LLO (LLO_T515AL516A_) revealed that a functional LLO was essential for activation of ERK1/2 in Caco-2 cells. More importantly, our previously identified LLO mutant (LLO_N478AV479A_) was unable to lyse erythrocytes within a certain concentration range (< ~15 nM), while becoming fully hemolytic at concentrations >20 nM. This change in cytolytic activity was neatly illustrated by the effect of the mutant on phosphorylation of ERK1/2; LLO_N478AV479A_ was incapable of inducing ERK1/2 phosphorylation at a low concentration of 5 nM, but the impaired ability could be fully restored by increasing the protein concentration to 20 nM. In addition, infection of human epithelial cells by *L. monocytogenes* synthesizing LLO_N478AV479A_ efficiently triggered ERK1/2 signaling. We have previously demonstrated that *L. monocytogenes* expressing LLO_N478AV479A_ was highly attenuated but still capable of growing intracellularly in macrophages and spreading cell-to-cell. Moreover, cytotoxicity of this mutant was as low as the wild-type strain (4). To date, the underlying mechanism of N478V479 in contributing to the pore-forming activity of LLO remains unclear. These two residues locate very close to the CDCs highly conserved undecapeptide (ECTGLAWEWWR), which was originally thought to be critical for cholesterol-mediated membrane recognition, as mutations in it abolished pore formation (41). However, it has recently been demonstrated that the undecapeptide was not directly responsible for cholesterol binding. Instead, a threonine–leucine pair in the C-terminal part of the protein was important (31). In fact, the conserved undecapeptide was shown to be a key structural element that allows the correct conformation of the cholesterol-binding motif (42), and the Trp-491 and Trp-492 within the motif have an important role linked to cathepsin-D in *Listeria* triggered innate immunity (43, 44). However, according to our previous study, the N478V479 play an important role in regulating the activity of LLO to minimize harm to host cells and thus establish a successful infection for this pathogen. Based on this, we therefore proposed a model suggesting that the moderate cytotoxic activity of LLO is essential for induction of MAPK ERK1/2 phosphorylation during host infection.

While pore-forming toxins allow pathogens to access the host cytosol, their activity must be tightly controlled and compartmentalized to avoid killing the host cells. The PEST-like sequence providing LLO with its uniqueness contributes to the pathogenesis of *L. monocytogenes* by making the LLO a phagosome-specific cytolysin with minimized cytotoxicity (5). The Portnoy group have shown that deletion of this sequence from LLO does not affect its pore-forming activity, but it does result in an avirulent phenotype. However, they showed that the PEST-like sequence appears to control the production of LLO in the host cell cytoplasm, but it does not promote its degradation (30). We here confirmed that removal of the PEST-like sequence had very minor effects on LLO pore-forming activity (Figure 4D) and consequently did not affect the property of LLO-mediated ERK1/2 phosphorylation (Figure 4E). Together with previously published data, the findings from this study firmly support the view that *L. monocytogenes* has evolved multiple sophisticated mechanisms to minimize harm to host cells by regulating the activity of LLO. To establish a successful infection and achieve maximal virulence, this pathogen must maintain an equilibrium between producing LLO that is cytolytic enough to mediate escape from the vacuole and sufficient to trigger a variety of cellular responses, yet is not overly toxic to infected host cells (45).

The ERK1 and ERK2 pathway has important roles in macrophages, regulating cytokine production via both transcriptional and post-transcriptional mechanisms. ERK1/2 activation downstream of TPL2, the tumor progression locus 2 (MAP3K), has complex effects on inflammatory responses, inducing the production of TNF, IL-1β and IL-10 following Toll-like receptors (TLR) stimulation, but negatively regulating the production of IL-12, IFN-β and iNOS (46). Considering the importance of MAPK signaling pathways in regulating immune responses, it is not surprising that many bacterial pathogens have developed sophisticated mechanisms to directly modulate MAPK activation to propagate infection (24, 26). One mechanism used by pathogens to modulate MAPK signaling is to interfere with the phosphorylation of MAPK cascades. The virulence of *Mycobacterium tuberculosis* is inversely related to its ability to activate MAPK signaling. This correlation has been linked to altered expression of the acetyltransferase, enhanced intracellular survival protein (Eis) by acetylating amino terminal lysine residues in the MAPK-docking domain of DUSP16 (47). *Yersinia* spp. employ the type III secretion system effector protein YopJ to inhibit MAPK by acetylating key residues in the activation loops of MAP kinase kinases (MKKs), which prevents their phosphorylation and consequently blocks the activation of MAPKs (48). Two other bacteria with type III secretion systems, *Salmonella enterica* and *Vibrio parahaemolyticus*, also block MAPK activation using acetylases to prevent MKK activation (49, 50). Collectively, as we reviewed previous studies on the modulation of MAPK signaling by bacterial pathogens, most of the pathogens were found to have developed mechanisms to directly inhibit MAPK activation for their own gain (24). However, one exception is the pathogen *Salmonella enterica* serotype Typhimurium (*S. Typhimurium*) that uses the type III secretion effector SteC to promote actin cytoskeleton reorganization by activating a signaling pathway involving the MAP kinases MEK and ERK, thus contributing to controlling intracellular replication of this pathogen. The rate of intracellular growth of *Salmonella* appears to be a tightly controlled process involving a balance between the activity of replication-promoting and replication-restraining virulence proteins, and restraining bacterial proliferation during infection is also therefore very important for *Salmonella* virulence (51).

Interestingly, for *L. monocytogenes*, activation or inactivation of MAPK pathway signaling by *L. monocytogenes* infection of different host cell types plays a critical role in bacterial invasion and intracellular growth in host cells (13, 15, 22, 26). We suggest that *L. monocytogenes* has evolved sophisticated mechanisms to flexibly modulate host cell signaling under various infection conditions for its own gain. Moreover, previous studies have shown that the inflammasome was activated by *L. monocytogenes* during infection, and some researchers suggested that caspase-1 activation was LLO-dependent, and such activation of the inflammasome was mediated by the pore-formation activity of LLO on the plasma membrane (52). Although the mechanism employed by LLO to modulate MAPK signaling during *L. monocytogenes* infection remains elusive, based on these studies we speculate that the pore-forming activity of LLO plays a dominant role in linking LLO-mediated MAPK signaling and host immunity modulation.

In this study, LLO permeabilization activity on the host cell membrane is demonstrated as critical for activating *L. monocytogenes* infection-induced ERK1/2 phosphorylation, and adding the purified LLO alone at a very low cytotoxic concentration is sufficient to induce this cellular response. The PEST-like sequence is not required for LLO-mediated ERK1/2 phosphorylation during infection, while mutations in the cholesterol-binding motif of LLO render this cytolysin incapable of activating MAPK signaling. Our study generates new insights toward understanding the mechanisms employed by LLO that allow *Listeria* to trigger a wide range of host cell responses during infection.

## Data Availability Statement

The datasets generated for this study are available on request to the corresponding author.

## Ethics Statement

The animal study was reviewed and approved by Institutional Animal Care and Use Committee of Science Technology Department of Zhejiang Province (Permit Number: SYXK-2018-0010).

## Author Contributions

CC, JS, and HS conceived and designed the experiments and wrote the paper. CC, JS, HY, TM, HZ, CG, ZC, and XZ performed the experiments and analyzed the data. CC, JS, ZC, and HS acquired the funds. All authors contributed to the review of the manuscript.

## Conflict of Interest

The authors declare that the research was conducted in the absence of any commercial or financial relationships that could be construed as a potential conflict of interest.
